# A Short Review of Membrane Fouling in Forward Osmosis Processes

**DOI:** 10.3390/membranes7020030

**Published:** 2017-06-12

**Authors:** Youngpil Chun, Dennis Mulcahy, Linda Zou, In S. Kim

**Affiliations:** 1 Natural and Built Environments Research Centre, School of Natural and Built Environments, University of South Australia, Mawson Lakes 5095, Australia; Dennis.Mulcahy@unisa.edu.au; 2 Singapore Membrane Technology Centre, Nanyang Environment & Water Research Institute, Nanyang Technological University, Singapore 637141, Singapore; 3 Department of Chemical and Environmental Engineering, Masdar Institute, Khalifa University of Science and Technology, Masdar City, P.O. Box 54224, Abu Dhabi, UAE; 4 Global Desalination Research Center, School of Earth Science and Environmental Engineering, Gwangju Institute of Science and Technology, Gwangju 61005, Korea; iskim@gist.ac.kr

**Keywords:** forward osmosis, membrane fouling, fouling monitoring, membrane cleaning, membrane surface modification

## Abstract

Interest in forward osmosis (FO) research has rapidly increased in the last decade due to problems of water and energy scarcity. FO processes have been used in many applications, including wastewater reclamation, desalination, energy production, fertigation, and food and pharmaceutical processing. However, the inherent disadvantages of FO, such as lower permeate water flux compared to pressure driven membrane processes, concentration polarisation (CP), reverse salt diffusion, the energy consumption of draw solution recovery and issues of membrane fouling have restricted its industrial applications. This paper focuses on the fouling phenomena of FO processes in different areas, including organic, inorganic and biological categories, for better understanding of this long-standing issue in membrane processes. Furthermore, membrane fouling monitoring and mitigation strategies are reviewed.

## 1. Introduction: Forward Osmosis (FO) as an Alternative Membrane Technology

**Figure 1:**
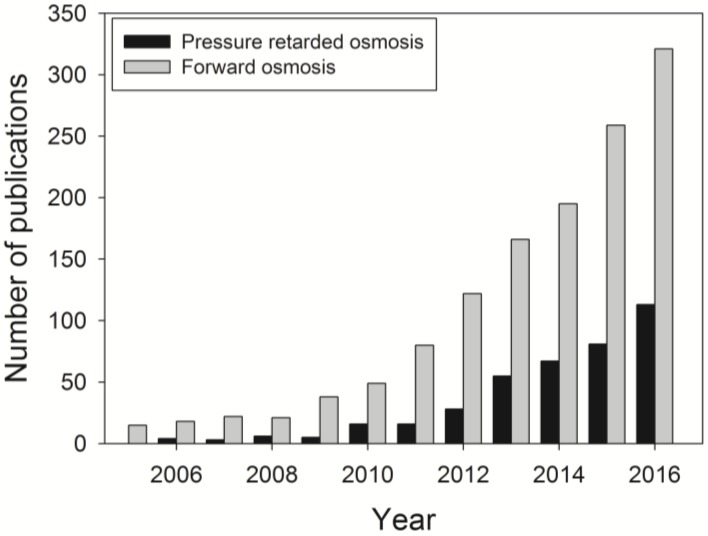
Increase in publications on forward osmosis between 2005 and 2016. The number of publications is calculated based on the Scopus database using keywords “forward osmosis” and “pressure retarded osmosis”.

As an emerging membrane technology, FO has attracted much research interest in the last decade [1,2,3]. The growing interest can be reflected by the increased number of academic publications on FO as shown in Figure 1. A total of 1306 and 394 academic documents were published on FO and pressure retarded osmosis (PRO) between 2005 and 2016, respectively. The FO process is a state-of-the-art membrane process that uses natural osmotic pressure as the driving force. This method is of considerable importance to efficient osmotic dilution processes or pre-treatments prior to various post-FO systems such as reverse osmosis (RO) desalination or membrane distillation, particularly treating challenging feed waters with high salinity and fouling potentials [4]. Whilst lots of information is available on fouling and cleaning of the membranes in pressurised membrane processes, the influence of organic-, inorganic- and bio-fouling on the membrane surfaces in wastewater treatment or desalination is still limited. In addition, since the FO process uses natural osmotic pressure as the driving force, the fouling and cleaning behaviour may be different from that in other pressure-driven membrane processes. The onset of fouling may result in reduced or improved treatment efficiency, depending on the intrinsic mechanisms, and will affect the effectiveness and applicability of FO as a pre-treatment for water purification [5]. While there are a number of comprehensive review articles available on FO [1,2,3,4,6,7], the purpose of this review is to concisely elaborate the cause, characterisation and alleviation techniques of FO fouling. Although the advantages of FO are the relatively lower fouling propensity and higher reversibility than the pressure-driven membrane processes [4,5,8], fouling is unavoidable and undesirable. Thus, better understanding of fouling mechanisms and fouling mitigation strategies in FO will be beneficial in various potential applications.

**Figure 2:**
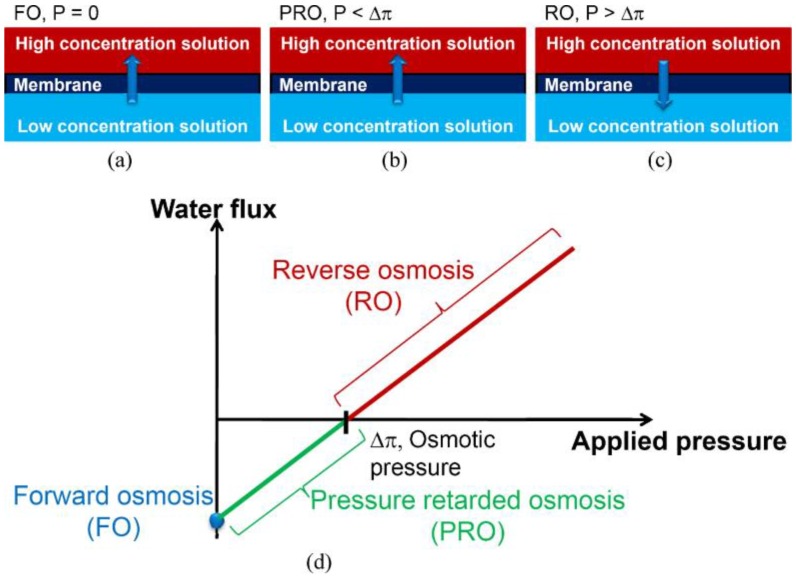
Illustration of FO, PRO, and RO processes: (**a**) FO process where no pressure is applied on the high concentration solution. Water flows from the low concentration side to the high concentration side; (**b**) PRO process where applied pressure on the high concentration solution is less than the osmotic pressure difference across the membrane. Water flows from the low concentration side to the high concentration side; (**c**) RO process where applied pressure on the high concentration solution is greater than the osmotic pressure difference across the membrane. Water flows from the high concentration side to the low concentration side; (**d**) Classification of FO, PRO, and RO in a flux versus pressure plot. Reprinted from [1,25,26], with permission from Elsevier.

FO systems exploit the natural osmotic pressure difference resulting when a semipermeable membrane separates two solutions with different concentrations. These are referred to as a feed solution (FS) of higher water chemical potential (lower osmotic pressure) and a draw solution (DS) of lower water chemical potential (higher osmotic pressure) [9]. During this process, the fresh water molecules move across the membrane and the DS is diluted. FO systems use an osmotic pressure gradient as the driving force, thus there is no externally applied hydraulic pressure and hence no cake layer compaction (Figure 2a). There are other advantages compared to other membrane processes, including high solute rejection and relatively low fouling propensity [10]. Novel FO membranes have been used for wastewater reclamation [11,12], brackish groundwater desalination [13,14], and seawater desalination [15,16,17], power generation [18,19,20], food processing [21,22], and production of fertiliser [23,24]. The application of these FO techniques has shown promising outcomes. The PRO process is similar to the FO process, except that an additional back pressure is applied in the DS (Figure 2b). Water permeation occurs as long as the applied pressure is lower than the osmotic pressure across the membrane. The PRO process also utilises the osmotic pressure (water chemical potential) gradient between seawater, or concentrated brine, and fresh water with pressure applied to saline stream. The water flux generated by the osmotic pressure of seawater is thereby converted into a hydrostatic pressure (mechanical energy) that can be used to produce electricity [1]. When the applied pressure is greater than the osmotic pressure difference, the direction of the water flux is reversed, which is the well-known RO process (Figure 2c) [25]. The flux versus pressure relationship in each process is illustrated in Figure 2d.

One of the key factors in developing a FO membrane is selecting an appropriate DS [1]. The ideal DS should generate high osmotic pressure and be inexpensive, non-toxic to the environment, and efficiently regenerated [27,28]. Often sodium chloride is selected as the draw solute as it has high water solubility and is relatively easy to reconcentrate using conventional desalination processes (e.g., RO or distillation) with minimal scaling [1]. Recent studies have used various DSs such as low-molecular-weight salts (NaCl, MgCl_2_, CaCl_2_, KCl, MgSO_4_) [1], sucrose [29], glucose [30], 2-methylimidazole-based compounds [31], magnetic nanoparticles [32], thermolytic inorganic salts (e.g., ammonia-carbon dioxide) [33], and hydrogels [34] that allow the creation of high osmotic pressure and can be regenerated or recovered. However, their costs have not been successfully established [35]. To produce purified water and sustain the driving force in the FO process, continuous reconcentration of the DS is required (e.g., by RO and distillation or a closed loop system) [36], which can be highly energy intensive [25], so that reconcentration or replenishment of the DS is not economical. If the draw solute is prejudicial to the aquatic environment, an additional treatment step is required prior to discharge [9]. The drawback of application of a costly draw solute can be solved by integration with a seawater desalination system or wastewater reclamation. Either seawater or brine from a spiral-wound reverse osmosis (SWRO) desalination plant can be utilised as the DS [37]. The seawater and/or brine are diluted with an impaired water source (e.g., treated wastewater effluent) and sent to the desalination RO stage or discharged to the ocean, respectively. Dilution of seawater reduces the required hydraulic pressure in the RO stage and hence, the energy demand for desalination, while dilution of brine lessens adverse impacts of desalination plants on marine environments [4,37]. Successful application of the osmotic dilution process will significantly improve the efficiency and sustainability of desalination or wastewater treatment processes [4,38].

## 2. Mass Transport in FO

**Figure 3:**
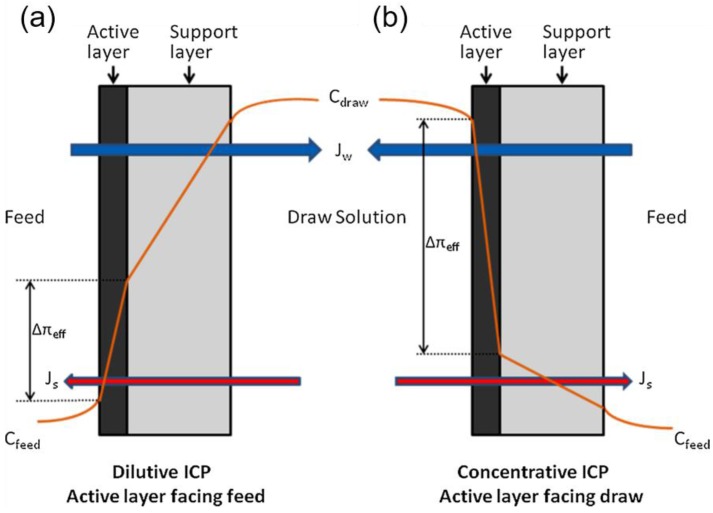
Illustration of both internal concentration polarisation (ICP) and external concentration polarisation (ECP) through an asymmetric FO membrane in (**a**) active layer facing the feed solution (AL-FS) and (**b**) active layer facing the draw solution (AL-DS) orientations. ICP occurs within the membrane support layer, and ECP exists at the surface of the membrane active layer. *C_feed_*, *C_draw_*, $\Delta \pi_{eff}$, *J_s_* and *J_w_* represent the feed solution concentration, draw solution concentration, effective driving force, reverse salt flux and water flux, respectively. Reprinted from [40] with permission from Elsevier.

Despite the possible new approaches as a pre-treatment combined with RO or distillation and the benefits of using a FO process, FO still has limitations, such as relatively low water flux, solute diffusion from the DS into the FS (and vice versa), concentration polarisation (CP), and membrane fouling [25,36]. Two factors significantly impeding osmotically-driven membrane filtration performance are CP and reverse permeation of draw solutes. CP is known to significantly reduce the membrane performance due to the reduced concentration gradient across the membrane rejection layer [39]. Theoretically, a semipermeable membrane would reject any dissolved draw solute into the FS. However, practically, small amounts of dissolved solute will be transported across the membrane. Reverse salt transport not only reduces the driving force, but may also enhance membrane fouling, and have inhibitory or toxic effects on the microbial community in the membrane bioreactor (MBR) systems [11]. Therefore, it is essential to understand the mass transport in osmotically-driven membrane systems to understand fouling. In FO, the water from the FS of lower concentration (higher water chemical potential) is transported through the semi permeable membrane into the DS of higher concentration (lower water chemical potential). At the same time, the draw solutes diffuse into the FS in an opposite direction to the water permeation [3]. This is defined as reverse salt diffusion (RSD). The rates of water permeation and RSD are affected by CP: internal concentration polarisation (ICP) occurs within the membrane porous layer, and external concentration polarisation (ECP) exists at the surface of the membrane’s active layer–bulk fluids interface during the mass transport in FO. It significantly reduces the effective osmotic driving force of the FO and the best known factor affecting membrane fouling, respectively (Figure 3) [36]. All membrane processes suffer from ECP effects at the membrane interfaces that are in contact with the bulk fluids, because a thin layer of fluid at the interface can become polarised. Within this thin layer of fluid, transport of water and other solutes is only based on advection (perpendicular to the membrane surface) and molecular diffusion [30]. The ICP effect exhibits a more severe impact on the reduction of water flux in the FO process than the ECP effect due to the fact that there is also an axial flow of salt solution within the porous layer of the asymmetric FO membrane. The solutes that enter and exit the porous layer are brought into the membrane surface layers by advective water flux and direct diffusion, and since only a minimal amount of solute can penetrate the dense selective layer, this will result in back diffusion and the build-up of solute within the porous layer leading to the formation of the ICP effect [30].

The mass transport across a membrane in the FO orientation can be expressed by the following equation [41]:

(1) $$J_{w}=A\left\{\frac{\pi_{d,b}\exp\left(-\frac{J_{w}\times S}{D}\right)-\pi_{f,b}\exp\left(-\frac{J_{w}}{k}\right)}{1+\frac{B}{J_{w}}\left[\exp\left(\frac{J_{w}}{k}\right)-\exp\left(-\frac{J_{w}\times S}{D}\right)\right]}\right\}$$

where $J_{w}$ is the water flux, *A* is the water permeability, $\pi_{d,b}$ is the osmotic pressure of the draw solution, S is the structural parameter, D is the draw solute diffusivity in water, $\pi_{f,b}$ is the osmotic pressure if the feed solution, k is the feed solute mass transfer coefficient and B is the solute permeability. The structural parameter S is often defined as S=tτ/ε, where t is the thickness of the support layer, τ is the tortuosity, and ε is the porosity. The *S* value is an important intrinsic membrane parameter. It directly affects the level of ICP and as such the flux though the membrane. This means that a thin, porous open structure, and low tortuosity is preferable for FO over thick tortuous membranes [42].

Normally, two types of membrane orientations can be employed for FO processes: the active layer facing the feed solution (AL-FS) orientation (FO mode) and the active layer facing the draw solution (AL-DS) orientation (PRO mode). Tang et al. [10] systematically investigated the effect of ICP during filtration of humic acid as a model foulant and discovered that more severe ICP was observed in the AL-FS orientation compared to the AL-DS orientation as a result of more severe ICP in the FO support layer than for the alternative membrane orientation. However, the AL-FS orientation offers stable flux against dilution of the DS and membrane fouling [10,43]. In this configuration, any attempts to decrease the flux are compensated by the reduced ICP. As a result, there is only a marginal flux decline at the expense of harsh initial ICP. In addition, foulant deposition is insignificant in this membrane orientation. In contrast, the AL-DS orientation exhibits severe flux decline due to: (1) the internal clogging of the support layer; and (2) the resulting enhanced ICP in the support layer.

RSD has also been one of the challenges in FO. The RSD from the FO DS can influence the FS chemistry such as ionic strength, pH and specific ionic concentrations, which possibly affect the fouling behaviours [3]. In order to reduce RSD, multivalent ions with lower diffusion coefficients are preferable as higher rejection of draw solutes can be achieved [1]. However, it is known that these ions (e.g., Ca^2+^ or Mg^2+^) may form molecular bridges with organic or colloidal foulants and accelerate membrane fouling [8,44]. In addition, due to their larger ion sizes, they may induce more severe ICP [40]. RSD from the FO DS was also found to be enhancing foulant-membrane interaction at the surface–water interface [45] or accelerating cake-enhanced osmotic pressure (CEOP) within the existing fouling layer [46].

## 3. Membrane Fouling in FO

A major drawback in any membrane water treatment system is fouling of the membrane surface. Various aspects of mass transport lead to the attachment, accumulation or adsorption of particles onto membrane surfaces and pores, causing membrane fouling [47]. Although membrane technology has advantages over conventional water treatment, membrane fouling continues to be a major operational problem [48]. Fouling of the membrane can occur as a result of a variety of contaminants in the feed water including inorganic compounds, colloidal or particulate matter, dissolved organics, chemical reactants, and microorganisms and microbial products [49,50,51]. However, it is considered difficult to predict the origin and extent of the effects of the simultaneous occurrence of these fouling mechanisms [47]. Although fouling can be reduced by employing low-fouling membrane materials, optimising the system operation conditions, and cleaning membrane units, it is unavoidable when feed water contains certain materials. Membrane fouling results in the requirement of pre-treatment of source water and membrane chemical cleaning, which incurs additional costs and increases energy consumption. Therefore, minimisation of the fouling is the key to success and cost-effective membrane operation [52]. The fouling mechanisms in FO systems may be different from and more complicated than those in the pressure-driven membrane processes [10,53]. For instance, Luo et al. [54] compared bacterial variation, contaminant removal and membrane fouling between an osmotic membrane bioreactor (OMBR)-RO and a conventional MBR-RO processes. Due to salinity build-up in the bioreactor, biomass characteristics and microbial community structure were altered, and soluble microbial product (SMP) and extracellular polymeric substances (EPS) in the mixed liquor increased in the OMBR. As a result, fouling was more apparent on the FO membrane in the OMBR compared to the microfiltration (MF) in the conventional MBR process where only minimal fouling occurred. However, the OMBR had a less adverse impact on the subsequent RO fouling, where a patchy fouling layer was observed in the OMBR-RO while a homogeneous cake layer was formed in the MBR-RO. The FO membrane effectively prevented foulant permeation into the draw solution, and reduced fouling in the downstream RO membrane during the OMBR-RO process, while substantial humic- and protein-like substance and inorganic salts were detected from the RO foulants in the conventional MBR. Understanding the fouling behaviour in the FO processes is particularly important because both sides of the FO membrane are in constant contact with impaired waters: the active layer with impaired water and the support layer with synthetic or natural DSs (e.g., seawater or brine). For instance, when a FO system was used to treat actual secondary effluent wastewater on the active layer and pre-filtered seawater from Red Sea was used as the DS, natural organic matter (NOM)-biopolymer fouling occurred on the active layer and transparent exopolymeric particle (TEP) fouling occurred on the support layer [55]. Therefore, there is a critical need for a systematic understanding of membrane fouling behaviour and for the development of strategies for fouling mitigation.

### 3.1. Organic Fouling

Organic fouling occurs by organic macromolecules found in the feed water. It can be caused by hydrophobic, transphilic and hydrophilic fractions in the feed water. Some studies have reported that the hydrophobic fractions (e.g., humic acids) are the main source of fouling in membrane filtration, but other research has indicated that hydrophilic (e.g., polysaccharide) fractions are the major problem [56,57]. Another study documented that the adsorption tendency of the polysaccharides on membrane surfaces is three times higher than that of humic acids [58]. For wastewater treatment using the membrane bioreactor (MBR) application, organic fouling is the major issue where organics are the precursor of biofouling. This is the dominant foulant in MBR applications [59]. It is also known that the concentration of the organic matter in seawater is relatively low (about 1–3 mg/L) and accordingly the portion of organic foulants is small in comparison with inorganic constituents. However, seawater organic matter is a more severe problem to be solved especially in SWRO, as it can also be converted to biofouling [60]. For the prevention of biofouling in membrane-based water treatment processes, control of organic fouling is essential to sustain water purification without hampering the overall system performance. In general, hydrophilic, H-bond acceptor, non-H-bond-donor, and neutrally-charged membranes are resistant to organic fouling [61], while hydrophobic and rougher membranes are prone to fouling by NOM and SMP [62].

**Figure 4:**
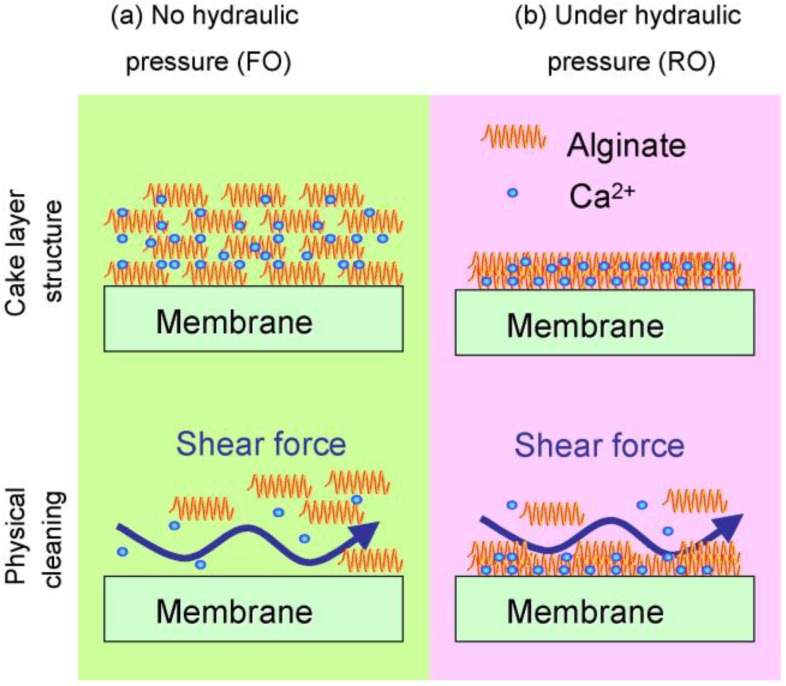
Schematic illustration of the fouling and cleaning with and without hydraulic pressure. (**a**) Loose alginate fouling layer due to the lack of hydraulic pressure, which permits effective physical cleaning; (**b**) Compact alginate fouling layer formed under hydraulic pressure, which results in low cleaning efficiency. Reprinted from [5], with permission from Elsevier.

In FO processes, organic fouling has been extensively studied in the last decade [5,8,14,55,63]. Mi and Elimelech investigated organic fouling in FO processes [8]. They found a strong correlation between organic fouling and intermolecular adhesion force, indicating that foulant–foulant interaction plays an important role in determining the rate and extent of fouling. Atomic force microscopy (AFM) adhesion force measurement also demonstrated that a small percentage of adhesive sites on the membrane surface play an important role in organic fouling formation and in decreases in cleaning efficiency. It was found that permeation drag, hydrodynamic shear force, and calcium binding are the major factors governing organic fouling development. It was discovered that organic fouling (alginate as a model foulant) is reversible without chemical cleaning [5]. Although the flux patterns in FO and RO modes were similar, the flux recovery rate after cleaning in FO process was much higher than for RO process. It is likely that the fouling layer formed (more related to inorganic scaling) on the FO membrane surface will be less compact due to the lack of hydraulic pressure (lower flux than pressurised membrane system; Figure 4). Xie et al. [64] supplemented these findings by comparing the alginate fouling characteristics between FO, pressure-assisted FO (PFO) and RO. Two possible mechanisms of fouling layer compaction have been proposed, namely permeation drag force and compression of foulants. The variations in the permeation drag force were eliminated by employing the identical initial water flux, and the fouling layer thickness, volume and density were identified. The fouling layer thickness decreased in the order of FO, PFO and RO while the volume and density increased from FO, PFO to RO. It was concluded that the applied hydraulic pressure contributed one factor to compression of fouling layers to a significant extent. The two possible compaction mechanisms may occur simultaneously and reinforce one another, resulting in irreversible, dense and compact fouling layers in RO in this study, while the drag force was the only applied compressive force in the case of FO. However, a contradicting study was also reported [65]. Lack of applied pressure does not necessarily mean that the FO is an inherently low fouling membrane process. There could be other contributing factors to the lack of significant fouling such as the compensating effect of ICP acting under AL-FS orientation as mentioned earlier in Section 2, and the low operating flux of the FO process could also induce the threshold/critical flux for less fouling.

She et al. [63] systematically investigated membrane fouling in the PRO process. Alginic acid and humic acid were used as feed foulants and various draw solutes were used, including synthetic seawater and seawater brine, NaCl, CaCl_2_, and MgCl_2_. RSD of Ca^2+^ and Mg^2+^, CaCl_2_, MgCl_2_, and synthetic seawater brine caused significant flux decline, although the FS does not contain Ca^2+^ or Mg^2+^. Compared to Mg^2+^, Ca^2+^ has much faster interaction with alginate due to the faster RSD of Ca^2+^ ions, while Mg^2+^ ions strongly interact with humic acid. Increased DS concentration had an enhancing effect on the flux decline due to increased RSD and initial flux. Increased applied pressure showed decreased water flux but increased RSD. The decreased flux can mitigate fouling by the flux-dependent fouling mechanism, and increased RSD can exacerbate membrane fouling by the RSD-enhanced mechanism. The relative importance of these competing factors is strongly dependent on the types of draw solutes. Increased pressure mitigated alginate fouling for the NaCl DS, while facilitating alginate fouling for the CaCl_2_ DS, indicating that RSD enhanced fouling strongly interacted with feed foulants.

**Figure 5:**
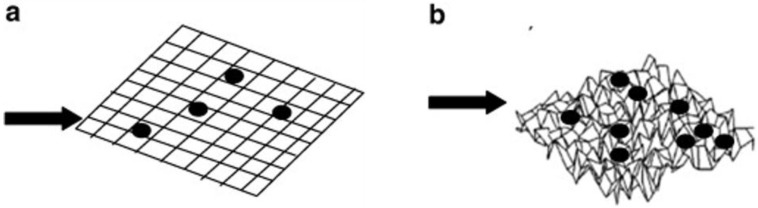
Illustration of (**a**) A smoother and non-porous FO membrane dense layer with less fouling; and (**b**) A rougher and more porous FO membrane porous layer with greater pore plugging and subsequent fouling. Reprinted from [66], with permission from Elsevier.

Parida and Ng [66] also studied organic fouling in different membrane orientations. More severe fouling was observed in the PRO mode due to the smoother and denser membrane layer in FO mode, whereby the roughness of the selective and the porous support layers was 66 and 105 nm, respectively (Figure 5). The loose structure of the porous support layer allowed the accumulation and deposition of foulants by the mechanism of direct interception and pore clogging, and facilitated further fouling development due to its rougher foulant surface and the foulant–foulant interaction. It was discovered that an organic loading of 50 mg/L—TOC (total organic carbon) or lower at a cross-flow rate of 50 cm/s caused minimal fouling in the FO mode during the 20 h of the fouling experiment. The presence of Ca^2+^ did not significantly deter the fouling resilience under this condition. It was suggested that FO mode is favourable when treating solutions with higher fouling/scaling tendencies (e.g., wastewater treatment) or higher salinity water (e.g., seawater desalination), while the PRO mode is to be preferred when using the solutions with lower fouling/scaling tendencies (e.g., brackish water desalination) or where intense concentration is necessary (e.g., power generation) [43].

### 3.2. Inorganic Scaling

Inorganic scaling occurs when the concentration of sparingly soluble salts such as calcium sulfate, barium sulfate, and calcium carbonate in the feed water exceeds their solubility at high product water recovery and, as a result, precipitation of these salts may occur near or on the membrane surface, leading to severe membrane flux decline [67]. Among the various scalants, calcium sulfate dihydrate (gypsum) and silica are the most common in seawater or brackish water desalination [67,68]. Mi and Elemelech [68] reported that flux decline rates in gypsum scaling experiments were practically the same in both FO and RO mode; however, more than 96% water flux recovery was shown in FO mode following a water rinse without chemical dosing. Flux recovery in RO mode was lower than in FO mode by 10%, which suggested that FO mode may provide the advantage of elimination of the need for a chemical agent for membrane cleaning. A similar study was conducted by the same authors [67] and the trends of water flux decline in silica scaling experiments were found to be similar in both FO and RO mode; however, almost 100% water flux recovery was shown in FO mode, and only 80% water flux recovery was obtained in RO mode. This indicates that although different driving forces (osmotic gradient and hydraulic pressure) did not result in different flux decline rates, they however may affect the structure or density of the formed scaling layer. When calcium ions are present with alginate (main component of polysaccharides) in the water, more pronounced flux decline is reported due to the formation of a cake/gel layer as described in the earlier subsection (Section 3.1).

In our recent pilot scale study on the fouling characterisation of a FO-RO system treating high fouling potential brackish surface water, inorganic scaling was found to be difficult to remove after the combination of physical and chemical cleaning [69]. Detailed characterisation revealed that it is likely gypsum and organic components presented in the FS could form the gel layer (calcium bridging) and enhance the fouling layer rigidity. A comparable study was published where seawater was treated using a spiral-wound FO (SWFO) module [70]. The fouling consisted mainly of scale-like foulants surrounded by biopolymeric substances. The silica scaling was caused by the polymerisation of dissolved silica. This silica scaling facilitated the deposition of NOM, as well as biopolymers. However, most NOM foulants can be easily removed while silica scaling is difficult to be physically removed. Work on a fertiliser-drawn FO (FDFO) process evaluated different types of draw solutes as a potential fertiliser while treating synthetic brackish groundwater for possible application of the process for irrigation [71]. Diammonium phosphate (DAP, (NH_4_)_2_HPO_4_) was observed to cause the most scaling among tested fertilisers as draw solutes due to their significant RSD phenomenon. Contrary to the previously mentioned studies, the flux decline caused by scaling was completely removed after physical cleaning with higher cross-flow velocity than the cross-flow velocity used for FDFO operation.

### 3.3. Biofouling

Biofouling is defined as the bacterial adherence with growth forming a biofilm, causing a membrane performance decline exceeding 10–15% of the start-up values under the applied operational conditions. At variations larger than 10–15%, corrective actions are recommended and guarantees are restricted by the manufacturers of membrane elements [72]. Biofouling causes significant technical problems and influences the system performance such as by increasing necessary operational pressure, bringing about membrane flux decline, causing membrane biodegradation leading to increased salt passage, and raising energy requirements. These eventually result not only in higher operating and maintenance costs but in a shortening of membrane lifetime. Produced water quality is also lowered.

In any membrane technology, biofouling control is considered as a major challenge because all other types of fouling are fairly readily avoided by either chemical and physical pre-treatment (viz. various inhibitors for inorganic scaling and physical pre-treatments for particulate fouling) [48,73]. However, biofouling formation only requires a few colonies to be developed with microorganisms present in all water systems which tend to adhere to surfaces and multiply on any surface in contact with the water even in an oligotrophic environment [74,75]. In addition, biofouling is a complicated process in which many factors can influence each other [72]. Biofouling is influenced by membrane surface properties (roughness, hydrophobicity, electrokinetic charge, and pore size), feed water chemistry (temperature, pH, ionic strength, nutrients, pollutants, and osmotic pressure) and also by microbial properties (size, cell surface hydrophobicity, and charge) [76,77]. Table 1 summarises the factors affecting microbial attachment to a solid surface.

Two major strategies are used to prevent biofilm formation. These are physical pre-treatment by MF or ultrafiltration (UF) of feed water and dosing with a biocide such as chlorine, respectively. Chlorination is considered as standard practice to control biofouling. However, it may generate harmful products such as trihalomethanes (THMs) and other potential carcinogens. In addition, chlorine shortens the membrane lifetime due to degradation and this leads to cost problems [74,79]. Physical pre-treatment applications have been extended to nutrient removal and modification of membrane surfaces to lower fouling occurrence [80]. Pre-treatment of the feed water may minimise microbial growth in the fouling layer. However, once attached on the membrane surface, microorganisms can grow and increase the amount of EPS [74,81]. Spiral-wound membrane elements, which are the most widely used in a variety of water treatment industries, make it difficult to remove the fouling layer due to their mechanical design (spacers, narrow feed channels, relatively low cross flow). Moreover, these spiral-wound designs directly support the accumulation of microorganisms and growth of biofilms [82].

It has been known that the EPS and SMP are the main fouling factors in membrane systems [83]. It is now recognised that TEPs play an important role in the process of aquatic biofilm formation [84], particularly in the early stage of biofilm development. Bar-Zeev et al. [84] introduced the new term ‘proto-biofilms’ to refer to TEPs with microbial outgrowth and colonisation. The authors found that these were the main sources of the early biofilm formation, particularly under the seawater condition.

Studies of biofouling in FO have been actively conducted recently, yet the understanding of this phenomenon is limited compared to other types of fouling. Further understanding of biofilm formation both in pressure-driven membrane systems and osmotically-driven membrane systems may help to develop strategies to control biofouling. Yoon et al. [85] studied biofouling occurrence by using the model bacterium *Pseudomonas aeruginosa* PA01 GFP in the FO process in comparison with the RO process and its control. They reported that biofouling is less significant in the FO process than the RO process. However, physical cleaning was not effective to overcome the water flux decline due to biofouling in the FO process, while chlorination effectively controlled the biofouling. Another approach of phosphate limitation in the FS to prevent microbial growth and biofilm formation was studied in a FO system treating wastewater [86]. They confirmed that limiting phosphate in the feed water is an effective way of inhibiting the water flux decline and biofouling development. In our previous study [14], significant biofilm deposition was observed on the FO membrane surface for nutrients-spiked brackish surface water filtration. Acute flux decline occurred at the initial stage of the experiment and significant decline was also observed at the end of the 24-h fouling experiment due to the formation of conditioning layers by organic foulants and the EPS biopolymer, respectively. The fouling experiments lasted for 40 h, and it was found that the declined flux was fully reversible by hydraulic rinsing using pure water only.

**Figure 6:**
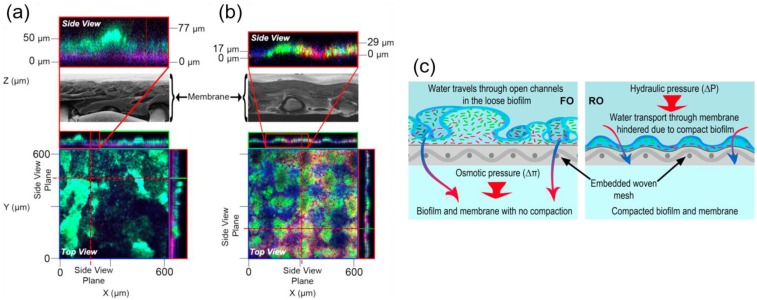
Confocal laser scanning microscopy (CLSM) orthogonal view of *Pseudomonas aeruginosa* biofilm structures developed on (**a**) FO and (**b**) RO membranes (scanning electron microscopy, SEM image) after biofouling for 24 h. The RO biofilm morphology followed the deformed membrane structure, as observed in the cross-sectional SEM images. The top insets are matching enlargements of the biofilm layer in side view; (**c**) Schematic illustration of biofouling formation in FO and RO processes, respectively. Reprinted from [87], with permission from Elsevier.

More recently, Kwan et al. [87] investigated the biofouling mechanisms in FO in comparison with the RO process using identical hydrodynamic conditions. The water flux decline was significantly lower in FO (~10%) than RO (~30%). Distinct differences in biofilm structure were observed. Biofilm characterisation using confocal laser scanning microscopy (CLSM) revealed that the FO biofilm exhibited a loosely organised thick layer (~50 μm) with prominent mushroom-shaped structure (~77 μm) while maintaining the initial conditions of membrane structure (e.g., finger like support layer structure) as shown in Figure 6a. This biofilm structure imparts low hydraulic resistance to water flow and low CP due to enhanced back transport of solutes to the bulk solution.

In contrast to FO, the RO membrane was compacted over the embedded supporting woven mesh due to the applied pressure, which was ~14.5 bar in this study. The biofilm formed on the RO membrane displayed a similar configuration that followed the shape of the membrane surface (Figure 6b). The biofilm exhibited a tight cell organisation (~29 μm), embedded in the polysaccharide layer. Interestingly, the live and dead cell components were slightly larger in FO, while polysaccharide concentration was higher in RO. The live to dead cell ratios for FO and RO were 0.72 and 0.52, respectively, which highlights the viability of the FO biofilm. This study concluded that applied hydraulic pressure creates a distinct difference in biofilm structure and facilitates hydraulic resistance, possibly intensifying the biofilm-enhanced osmotic pressure. This adversely affects membrane water flux (Figure 6c). FO biofouling showed advantages in terms of membrane cleaning and filtration of high fouling potential feed waters.

It should be noted that the combined fouling occurs simultaneously. A number of studies have investigated the effect of combined fouling in FO processes and reported their synergetic detrimental effects on the membrane performance [35,69,88,89,90,91,92]. Zhang and co-workers investigated the combined effect of organic fouling and inorganic scaling in a forward osmosis membrane bioreactor (FOMBR) [89]. The fouling in a FOMBR treating wastewater was governed by the coupled influence of biofouling and inorganic scaling in the AL-DS orientation, while AL-FS offered stable flux when combined with intermittent cleaning using tap water, suggesting that the AL-FS orientation should be recommended for FOMBR. Liu and Mi [88] also confirmed a synergetic effect of alginate fouling and gypsum scaling on FO performance. The aggravated gypsum scaling in the presence of alginate molecules caused more severe flux decline than the algebraic sum of flux declines by the individual foulant. In a detailed investigation of the effects of alginate on the kinetics of gypsum crystal growth, they revealed that alginate molecules act as nuclei for heterogeneous crystallisation of gypsum, resulting in a combined network of gypsum and alginate fouling. The alginate molecules shortened the nucleation time so that it increased the rate of gypsum growth and changed the morphology of gypsum crystals, also increasing their size.

## 4. Membrane Fouling Characterisation

It is important to determine the fouling characteristics in order to provide insights into pre-treatment and fouling mitigation strategies. Membrane fouling can be categorised into chemical, physical and microbiological. Surface charge and hydrophobicity via zeta potential and contact angle measurements, were widely used for chemical characterisation. Surface morphology (e.g., variations in the distribution of peak and valley structures), which is commonly determined by scanning electron microscopy (SEM) and AFM is often employed for physical characterisation [93]. Some of the common microbiological characterisation techniques include adenosine triphosphate (ATP) measurements, EPS quantification, and application of CLSM. Based on these surface characteristics, it has been found that the propensity for fouling increases for membranes that are highly charged, more hydrophobic, and rougher [94]. Fouling in pressure driven membrane-based water treatment systems is normally detected by trans-membrane pressure drop increase or reduction in permeate flux, and the diagnosis is performed by membrane module autopsy, followed by time and labour-consuming analytical techniques [72,95]. Due to the hindrance of the destructive method such as damage, contamination, and structural changes of the samples and variability of the small-scale samples, there is a substantial need for better tools and measurements which are in-situ, real-time, and non-destructive [95,96]. Since information on the widely used fouling characterisation methods is readily available, some of the new fouling monitoring and characterisation techniques are summarised in the following sub-section.

### Some Emerging Fouling Monitoring and Characterisation Techniques

Ultrasonic time-domain reflectometry (UTDR), as a membrane fouling monitoring tool, has been extensively studied [97,98,99,100,101]. The ultrasonic signal amplitude provides sensitive results on the dynamics of fouling layer growth that are comparable to those obtained from the flux decline patterns. The UTDR was also successfully employed for membrane cleaning studies. Fouling layer removal was monitored under ambient and high pressure conditions. The results of ultrasonic measurements correspond well with the membrane surface morphological characterisation studies [97,98]. More recently, Sim and co-workers have used UTDR for colloidal and biofouling detection [99,100]. The UTDR response corroborated well with the transmembrane pressure behaviours during fouling development and offline fouling characterisations.

**Figure 7:**
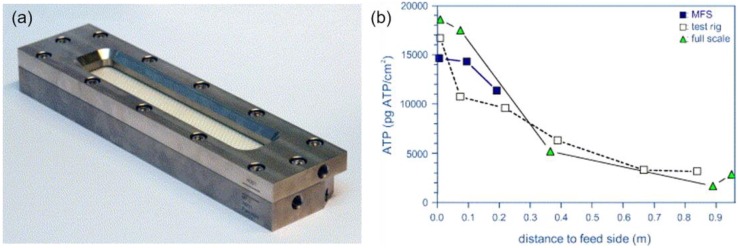
(**a**) A photograph of the developed membrane fouling simulator (MFS), external dimensions of 7 cm × 30 cm × 4 cm); (**b**) Biomass concentration over the length of the MFS in comparison with the test rig and full scale module. Reprinted from [95], with permission from Elsevier.

Vrouwenvelder et al. [95] developed a membrane fouling simulator (MFS) as an early warning tool for membrane fouling monitoring as shown in Figure 7a. The MFS showed the same development of pressure drop with time and concentration of active biomass with spiral-wound membrane modules (Figure 7b). The inlet port of the lead element was significantly fouled, and a decline was observed over the length of the spiral-wound membrane modules. This suggests that the MFS enables early warning of biofouling in full scale modules as the biomass accumulation occurred on the first 20 cm of the lead membrane module as shown in Figure 7b. In addition, the fouling development was observed through the transparent window and a sample (membrane coupon) from the MFS can be characterised for fouling identification.

**Figure 8:**
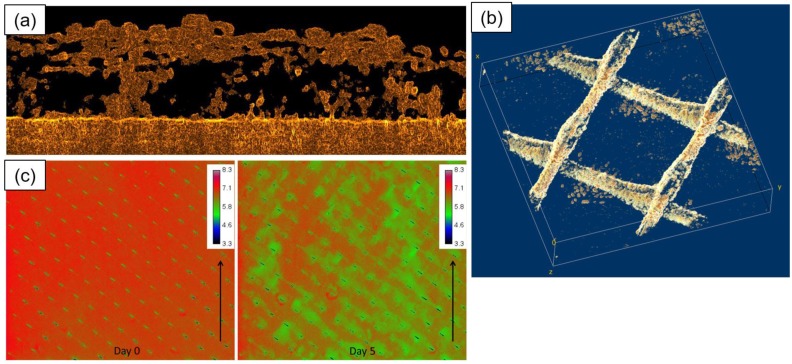
(**a**) Optical coherence tomography (OCT) 2-D image of a grown biofilm on a membrane surface without feed spacer in an area of a 3.83 mm × 0.85 mm. The membrane is shown at the bottom of the figure. The biofilm had a heterogeneous structure containing voids; (**b**) 3-D reconstruction of a biofilm grown on the surface of a membrane and feed spacer in a flow cell; the image was obtained after processing OCT 3-D scans in an area of 6 mm × 6 mm × 1.08 mm. Spacer filaments contained most of the biomass detected; (**c**) Spatial distribution of oxygen concentration (mg/L) at the inlet side of the MFS on day 0 and after 5 days of biofilm development. The arrow indicates the water flow direction. The scale bar represents oxygen concentration (mg/L). The imaged area is 4.0 mm × 3.5 cm. Biofilm accumulation started on the feed spacer. Reprinted from [96], with permission from Taylor & Francis Online.

Nuclear magnetic resonance (NMR) microscopy was used to study membrane biofouling [102]. It is shown that NMR was able to visualise the distribution of biofilm in the membrane module and its impacts on the hydrodynamics and mass transfer. It was found that a minimal growth of biofilm had a substantial impact on the flow field homogeneity. From the data observed in this study, the effective membrane surface area was also quantified. Optical coherence tomography (OCT) has been introduced as an emerging technique to analyse biofouling [72]. OCT is a non-destructive and contact-free micrometre-resolution biofilm visualisation technique. OCT does not require the addition of stains or signal enhancers that may affect the biofilm. Biofilm development and structural changes were observed and subjected to permeate flux variations. In a recent article by Valladares Linares et al. [96], OCT, planar optodes (for mapping O_2_ concentration in biofilm), and NMR were employed to observe real time biofilm formation and its interaction with hydrodynamics and mass transport (Please see Figure 8).

The above introduced fouling monitoring or characterisation techniques fulfilled most of the requirements of an early fouling warning tool representative of full scale modules in data accuracy and reproducibility, in-situ, in real-time, and with non-destructive fouling characterisations. Thus, they have the potential to be applied as a fouling prediction and control tool. Some other non-invasive fouling monitoring or characterisation methods include electrical impedance spectroscopy (EIS), radio activation analysis, computerised axial tomography (CAT), small-angle neutron scattering (SANS) and electrochemical methods. Others are constant temperature anemometry (CTA) and direct observation through the membrane (DOTM), laser triangulometry, refractometry, photosensors, video microscopy, fluorescence microscopy and particle image velocimetry (PIV) [103].

## 5. Membrane Cleaning Strategies

In order to achieve constant flux in a membrane water purification system, an appropriate cleaning technique is required. In the majority of bench scale FO fouling experiments which generally employ FO flat sheet membrane coupons, fouling and declined flux is more reversible than in other pressure driven membrane processes [1,2,5,14,43,104,105,106,107]. Flux recovery in the FO mode was much higher than in the RO mode in alginate fouling experiments under identical cleaning conditions, although the rate of membrane flux decline was similar in the two modes [5]. The fouling reversibility of FO was attributed to the less compact organic fouling layer formed in the FO mode due to the lack of hydraulic pressure as described in Section 3. A similar observation was made by the same authors in the gypsum scaling experiments [68]. Zhao and Zou [105] systematically investigated the physical cleaning efficiency in inorganic scaling experiments under different temperature conditions. It was found that the flux ratio for before and after the cleaning was 0.997, 0.849, 0.792 for 25, 35, and 45 °C, respectively. Although higher temperature induces higher initial water flux and overall water recovery, more compact crystals were deposited on the membrane surface thus adversely affecting membrane cleaning efficiency. Air-scouring showed great fouling and flux reversibility. Zhang et al. [108] characterised fouling layers using a CLSM in an FOMBR and found that little biofilm formed under high aeration, while low aeration resulted in significant biofouling. Valladares Linares et al. [55] found that ~90% of the flux recovery was achieved by using 15 min of scouring for NOM-biopolymer fouling on the active layer and TEP on the support layer. It is known that the existing TEP pre-treatment is ineffective [109], thus the authors compared the effectiveness of various chemical cleaning agents. Cleaning solution (1) contained 0.8% sodium ethylenediaminetetraacetic acid (EDTA) and 1% Alconox, a detergent composed of sodium dodecylbenzenesulfonate (10–30%), sodium carbonate (7–13%), tetrasodium pyrophosphate (10–30%), and sodium phosphate (10–30%). Cleaning solution (2) contained 1% sodium hypochlorite (NaOCl). For the removal of TEP fouling, 1% NaOCl was more effective than the complex cleaning solution (1) in their study. The results suggest that operating in FO mode may offer an unprecedented advantage in reducing or even eliminating the need for chemical cleaning.

It should be noted that when the membrane surface is covered with foulants, the organic matter and the attached microbes cause the membranes to become more hydrophobic and rougher. If the shear force is not strong enough, the membrane fouling resistance can be reduced, and eventually becomes fouled [108]. Thus, more intensive cleaning strategies need to be considered. Yoon et al. [85] reported that physical cleaning was not effective for biofouling control, while chemical cleaning using NaOCl was more effective. Martinetti et al. [13] investigated the cleaning efficiency in vacuum-enhanced direct contact membrane distillation (VEDCMD) and FO, treating brine containing scalants. A chemical cleaning solution consisting of 0.029 M disodium ethylenediaminetetraacetic acid (Na_2_EDTA) and 0.058 M sodium hydroxide (NaOH) was used for scale removal. In the VEDCMD system (custom-made membrane distillation system in the laboratory), the Na_2_EDTA cleaning effectively restored the flux to its initial level, however immediate onset of flux decline was observed after the chemical cleaning. A similar observation was made in the FO system. Although the water flux was restored to its initial level, faster water flux decline patterns were observed after physical cleaning compared to chemical cleaning. This suggests that physical cleaning is able to remove the scale from the membrane surface but not necessarily the deposited scale inside the membrane structure, which leads to faster flux decline after cleaning. The residual foulants that remained on the membrane surface likely provide sites for crystallisation of scaling and the onset of fouling development occurred after the cleaning. Concerns about the damage of membrane by high pH of the Na_2_EDTA cleaning solution were confirmed by NaCl rejection tests. The cellulose tri-acetate (CTA) RO membrane that was exposed to Na_2_EDTA solution for 15 h showed no rejection of NaCl. Although the Na_2_EDTA cleaning solution is effective in removing calcium sulphate scale, it is not recommended for use with membranes that have a narrow operating pH range. In our recent study, a pilot-scale FO-RO system was employed to treat foulant-spiked brackish surface water [69]. The water flux in the FO stage was significantly decreased when the feed solution was spiked with gypsum and organics. The system was thoroughly cleaned after ~155 h of operation using a combination of physical and chemical cleaning agents, followed by membrane autopsy studies. The majority of hard-to-be-removed foulant was found to be composed of inorganic scalants rather than organic, or biofoulants. The thorough cleaning was not able to fully restore declined flux. The flux was restored initially but dropped within an hour to the same level as when the module suffered severe fouling. Also, divalent calcium ions worsen organic fouling by formation of intermolecular bridges among organic foulant molecules and thus efficient chemical cleaning could only be achieved when the calcium ion bridge was eliminated. It was reported that fouling and declined flux in FO is more reversible than in other membrane processes, and the AL-FS orientation is recommended for treating difficult feedwaters. However, our study observed contrasting results from the reported data. In fact, very few studies have reported successful cleaning of the membrane that restored membrane performance in NF and RO spiral-wound membrane elements [110,111,112]. Therefore, it is suggested that further research work should be conducted for effective cleaning strategies such as air scouring for a longer period [113], intermittent air/water flush [114], feed-concentrate flow direction reversal [115], and cleaning at an early stage by monitoring fouling using tools listed in Section 4 that minimise the use of cleaning agents or development a biocide-free antifouling process that optimises nutrient-limitation techniques.

When utilising membrane technology in water purification processes, fouling and the need for subsequent chemical cleaning are often inevitable [116]. For membrane cleaning, normally a combined cleaning strategy is used, by first weakening the fouling matrix by the use of chemical cleaning agents and then removing the fouling layers by mechanical forces. Table 2 shows typical cleaning chemicals commonly used. However, continuous chemical cleaning application creates wastewater problems which can be costly and also the wastewater discharge must comply with the more stringent environmental regulations [74]. Therefore, it is critical to understand the foulant–membrane, foulant–foulant, and foulant–cleaning agent interactions at a molecular level in order to unravel the mechanisms of fouling and the subsequent development of effective methods of membrane cleaning. Li et al. [116] determined the adhesion forces between the foulants and the membrane surface and between the bulk foulant and the fouling layer. The interfacial force data was assessed alongside the NF membrane water flux measurements to elucidate the mechanisms of organic fouling and chemical cleaning. A strong correlation was obtained between the measured adhesive force and the fouling and cleaning behaviours of the membrane under various solution chemistries of ionic strength, divalent cation concentration and pH. This study discovered that the cleaning efficiency of the membranes was highly dependent on solution pH and the concentration of the chemical cleaning agent.

## 6. Membrane Modification for Enhanced Performance and Fouling Mitigation

Significant advances in membrane fabrication technology have been made, especially in surface modification, where membrane surface properties have been tailored toward reducing membrane fouling as well as enhancing membrane permeability [93]. Despite the increased efforts to understand fouling behaviour in FO, antifouling membrane fabrication still needs to be explored. The ideal FO membrane characteristics would be a dense ultra-thin active layer with high water permeability and low solute permeability, which is supported by a relatively thin and highly porous substrate for low ICP. In addition, the membrane needs to be hydrophilic to help reducing membrane fouling, and possesses high mechanical strength to sustain long-term operation [118].

Various studies have reported the successful fabrication or modification of FO membranes. McCutcheon and Elimelech [119] examined the influences of polyester (PET) non-woven and polysulfone (PSF) support layers on cellulosic and thin film composite (TFC) RO membrane performance in FO cross flow experiments. Removal of the PET layers with the aid of RO pre-treatment or addition of surfactant in order to increase the wetting property of the membrane increased the water flux in cellulosic RO membranes, especially in the PRO applications, while very small changes were observed in TFC RO membrane function. As described in Section 2, ICP hinders any asymmetric membrane performance in FO [39]. In order to minimise the performance-limiting factors of ICP, the support layer should be very thin, highly porous, and provide a path from the DS to the active layer of the membrane [33]. Innovative membrane fabrication has been successfully carried out which fulfils these requirements [120]. The membrane active layer was formed by interfacial polymerisation on top of a PSF support layer fabricated by phase separation onto a thin polyester nonwoven fabric. The cast TFC FO membrane showed the highest water flux values compared to the commercial CTA FO and TFC RO with or without the PET layer, while maintaining great salt rejection performance (~97.4%). FO membranes with thinner, more porous and less tortuous support layers have smaller values of the structural parameter (*S*) and result in higher water fluxes. The *S* value for the TFC FO membrane was significantly reduced in comparison to other tested membrane samples. This substantially reduced ICP.

It is suggested that tubular or hollow fibre membranes may be more suitable for FO than the flat sheet membranes. It is much simpler to fabricate hollow fibre modules with a high packing density. They have a self-supporting structure, and possess the necessary flow patterns for FO [118]. TFC FO hollow fibres with an ultra-thin RO-like selective layer (300–600 nm) on a porous hollow fibre substrate (75–84% porosity) were fabricated by a two-step process: a phase inversion step for the hollow fibre structure and an interfacial polymerisation step for the RO-like skin layer [118]. The hollow fibre membranes showed great performance in intrinsic separation properties such as water permeability coefficient (*A* value) and salt (NaCl) permeability coefficient (*B* value) even though the *S* value was comparable with that of the flat sheet FO membrane. The fabricated membranes showed superior water flux values to the commercial membranes due to their hydrophilic property (43° contact angle) and excellent intrinsic separation performances—lower salt flux to water flux ratio—thus reducing the ICP effect.

Some practical and facile techniques were also reported for mitigation of fouling. Valladares Linares et al. [121] investigated the impact of spacer thickness (28, 31, and 46 mil) on biofouling in FO. The same amount of biomass accumulation was detected, while the flux reduction decreased with the thicker spacer. Not only the decrease in membrane thickness, but also hydrophilic chemistry obtained from polymers or hydrophilic additives, may also improve FO performance [122]. Nguyen et al. [123] evaluated the incorporation of a redox functional amino acid 3-(3,4-dihydroxyphenyl)-l-alanine (l-DOPA) onto commercial FO membranes to create a zwitterionic surface that resists membrane fouling. The top layer of the membrane surfaces was modified by the deposition of l-DOPA from an alkaline solution. The hydrophilicity of the coated membranes was significantly improved and a systematic increase in water flux was observed for the samples coated for up to 12 h. The coated membranes showed an improvement in their fouling resistance when testing with feed water containing alginic acid sodium salt solution. More recently, Liu et al. [124], successfully grafted TFC FO membranes using zwitterionic polymer (poly(sulfobetaine methacrylate)) in order to create a polymer brush via atom-transfer radical-polymerisation (ATRP). AFM results showed that membrane–foulant interaction forces significantly decreased by one order of magnitude after the surface modification compared to that of the pristine FO membrane. This study also demonstrated that great organic and biofouling mitigation was achieved after the membrane modification due to increased hydrophobicity and reduced surface roughness, while maintaining the intrinsic separation properties.

Nguyen et al. [125] extended the membrane modification by applying a photoinduced growth approach using silver (Ag)/titanium dioxide (TiO_2_). It was reported that the anti-bacterial action on the Ag/TiO_2_-coated FO membrane was significant compared to the virgin membrane (by ATP measurements). This was primarily due to the anti-bacterial effects of the silver nanopoarticles (AgNPs). The TiO_2_ played an effective role in regenerating the AgNPs by decomposing the organic matter that covered them. After the cleaning process, it was observed that 67–72% recovery of initial water flux was achieved by the Ag/TiO_2_-coated membranes, whereas only 33% flux recovery was achieved by the original membrane. Carbon-based nanomaterials such as carbon nanotubes (CNTs) and graphene oxide (GO) have also been extensively researched for enhanced membrane performance and antifouling properties. GO and GO-silver nanocomposite (GOAg) were functionalised to the polyamide active layer of the TFC membrane surface in a very recent study [126]. GO and GOAg nanocomposites were covalently bound to the TFC FO membrane via a crosslinking reaction. The authors confirmed that the intrinsic transport properties were not affected by the GO and GOAg linkage. In addition, GOAg-FO membrane significantly reduced the attachment of *Pseudomonas aeruginosa* cells and flux decline due to biofouling development, suggesting that the GOAg is a robust platform to yield enhanced biofouling resistance in a membrane fabrication process.

## 7. Concluding Remarks

FO has been extensively studied and applied in various water purification processes, food processing, power generation, and the pharmaceutical sector. However, the longstanding issue of membrane fouling hinders overall system performances in FO, as it does in pressure-driven membrane processes. Thus, it is essential to understand fouling mechanisms and their mitigation strategies, including effective cleaning methods and anti-fouling membrane fabrication for sustainable FO operation. Since the information related to fouling in FO processes is still limited, in-depth and systematic study is needed. This review explores FO fouling mechanisms in various categories including organic, inorganic, and biological sources and cleaning implications. Based on this review, the authors have discussed some further insights into the understanding of membrane fouling mechanisms and their mitigation strategies in FO processes.

## Figures and Tables

**Table 1:** Factors contributing to microbial attachment. Adapted from [78]. EPS: extracellular polymeric substances.

Substratum	Bulk Solution	Cell
Hydrophobicity	Presence of anti-microbial chemicals	Cell surface hydrophobicity
Roughness	Nutrient availability	Extracellular appendages
Charge	Ionic strength	EPS
Porosity	pH	Species
Conditioning film	Temperature	Surface charge
Surface chemistry	Shear force	Growth phase

**Table 2:** Major chemicals for membrane chemical cleaning. Adapted from [117]. EDTA: sodium ethylenediaminetetraacetic acid.

Category	Chemicals	Functions
Alkali	NaOH	Hydrolysis, dissolution
Acids	Citric acid, nitric acid	Dissolution
Chelating agents	EDTA	Chelation
Oxidants	NaClO, H_2_O_2_, peroxyacetic acid	Oxidation, disinfection
Surfactants	Detergents, surfactants	Emulsifying, surface conditioning
